# P-596. Pneumococcal Burden and Serotype Distribution for Invasive Disease among High-Risk Population in Latin America and the Caribbean: a Systematic Review and Meta-analysis

**DOI:** 10.1093/ofid/ofaf695.810

**Published:** 2026-01-11

**Authors:** Ariel Bardach, Silvina Ruvinsky, Macarena Roel, Tomas Alconada, Carla Voto, Martin Brizuela, Paula Gagetti, Agustin Ciapponi

**Affiliations:** IECS, Ciudad Autónoma de Buenos Aires, Ciudad Autonoma de Buenos Aires, Argentina; Hospital de Pediatría Dr. Juan P. Garrahan, buenos aires, Ciudad Autonoma de Buenos Aires, Argentina; Hospital de Pediatria JP Garrahan, Buenos Aires, Ciudad Autonoma de Buenos Aires, Argentina; IECS, Ciudad Autónoma de Buenos Aires, Ciudad Autonoma de Buenos Aires, Argentina; Hospital de Pediatria Juan P Garrahan, Ciudad Autónoma de Buenos Aires, Ciudad Autonoma de Buenos Aires, Argentina; Hospital General de Agudos Velez Sarsfield, Buenos Aires, Ciudad Autonoma de Buenos Aires, Argentina; INEI-ANLIS "Dr. Carlos G. Malbran", Ciudad Autónoma de Buenos Aires, Buenos Aires, Argentina; IECS, Ciudad Autónoma de Buenos Aires, Ciudad Autonoma de Buenos Aires, Argentina

## Abstract

**Background:**

Data regarding invasive pneumococcal disease (IPD) burden, circulating serotypes and use of resources in high-risk populations (immunocompromised hosts, adults ≥65 y, and children < 5 y) in Latin America and the Caribbean (LAC) is scarce

**Methods:**

We performed a systematic review and meta-analysis, registered in PROSPERO CRD42025629682, following Cochrane, MOOSE, and PRISMA guidelines. MEDLINE, Embase, LILACS, regional surveillance reports, grey literature, and conference proceedings between 01/01/2000 and 03/31/2025 were searched. Two reviewers independently screened titles/abstracts and full texts, and assessed the risk of bias of included studies. Conflicts were resolved by consensus, and data on population, clinical setting, IPD incidence or prevalence, serotype distribution, case-fatality ratio (CFR), and healthcare utilization were extracted. Random-effects meta-analyses estimated pooled incidence rates and CFRs; serotype coverage by available and pipeline PCVs were summarized. Full pooled estimates and stratified analyses by age, risk group, CFR, use of resources were analized

**Results:**

Of 6,136 unique records screened, 198 studies from 18 LAC countries met inclusion criteria, 60% were pediatric cohorts including children < 5yo, 25% persons with immunocompromising conditions (mainly HIV infection or solid-organ transplantation), and 15 % older adults >65yo. Preliminary results indicates wide heterogeneity in reported IPD incidence (12–170 cases/100,000 person-year) and CFRs (4–29%). Serotypes 14, 19A, 6B, 3, and 23F, represented >60% of typed isolates during all periods. During the last 10 years a significant increase of non-vaccinal serotypes in all region was observed. Coverage of these serotypes by PCV13 averaged 77% (95% CI 71–83%) and is projected to increase theoretical coverage with higher-valent PCVs (PCV15/20/21)

**Conclusion:**

These findings provide an updated evidence base to support national immunization programs and guide future serotype-specific surveillance in the region

**Disclosures:**

All Authors: No reported disclosures

